# Outcome of multimodality treatment of Ewing’s sarcoma of the extremities

**DOI:** 10.4103/0019-5413.69307

**Published:** 2010

**Authors:** Akshay Tiwari, Himesh Gupta, Sandeep Jain, Gauri Kapoor

**Affiliations:** Department of Surgical Oncology, Rajiv Gandhi Cancer Institute and Research Center, Rohini, Delhi, India; 1 Department of Pediatric Oncology, Rajiv Gandhi Cancer Institute and Research Center, Rohini, Delhi, India

**Keywords:** Ewing’s sarcoma, extremity, multimodal chemotherapy

## Abstract

**Background:**

The management of Ewing’s sarcoma family of tumors (ESFT, Ewing’s sarcoma/primitive neuroectodermal tumor) has been established as a multimodality treatment. Advances in imaging and diagnostics, chemotherapy, surgical techniques, radiotherapy and prosthetic technology have resulted in drastic changes in the outcome of this disease, with most of the recent studies having 5-year survival rates of more than 60%. The Indian patients present at a more advanced stage and the compliance of treatment is suboptimal. While there is plenty of data in the world literature on the outcome of Ewing’s sarcoma, there is paucity of data in Indian patients. Therefore, we conducted the present study to analyze the outcome of multimodality treatment of ESFT of the extremities at a tertiary nonprofit institute over a decade.

**Materials and Methods:**

34 patients who had histopathologically proven diagnosis of Ewing’s sarcoma of the extremities and had received treatment at our institute from 1997 through 2007 were included for analysis. The majority of patients had involvement of the femur (35%), followed by tibia (17%), fibula and foot (15% each), humerus (12%) and soft tissue of thigh (6%). Twenty-nine patients presented with localized disease (Enneking stage II B) while five patients presented with metastases (Enneking stage III). All patients received Vincristine, Actinomycin D, Cyclofosfamide + Ifosfamide and Etoposide (VAC+IE)-based chemotherapy and local treatment was offered to all but three patients having multicentric disease. The local treatment offered were, radiation (n= 15), surgery (n= 12) both surgery and radiation (n=4). All patients were analyzed for oncological outcome (event-free and overall survival, local and systemic relapses) by clinical and imaging evaluation and functional outcome by using the musculoskeletal tumor society (MSTS) score. These outcomes were correlated with age, sex, size of tumor, stage at presentation, modality of local treatment and site of relapse.

**Results:**

At the final follow-up (mean, 26 months; median, 17 months; range, 3–97 months), the overall and event-free survivals were 47 ± 12% and 34 ± 9%, respectively. Sixty-two percent of the patients presented with a tumor size more than 8 cm. On correlation with age, sex, size of tumor, stage at presentation, modality of local treatment and site of relapse, no correlation of survival was seen with any of the variables except event-free survival with size of the tumor. The functional outcome of all the patients was satisfactory (MSTS score >16 out of 30). No patient underwent amputation.

**Conclusion:**

Although the demographic profile, stage at presentation and the local and systemic treatment regimen followed in our study was similar to the world literature, the outcome of Ewing’s sarcoma in Indian patients were found to be inferior to that reported in the western literature. Larger multicentric studies with longer follow-up are required to exactly determine the key areas crucial in improving this outcome.

## INTRODUCTION

Ewing’s sarcoma mostly affects the bone and soft tissue.^1^–^3^ However it has also been reported in other organs. The site of disease has been a major determinant of the oncological outcome of patients with Ewing’s sarcoma, with visceral involvement being a bad prognostic marker than bone and soft tissue. Among bone and soft tissue Ewing’s sarcoma, involvement of the extremities is reported to have a better prognosis than those located in the axial skeleton.^2^ The treatment of extremity Ewing’s sarcoma has undergone rapid change as a result of recent improvement in diagnostics, imaging, limb salvage surgery, prosthetic technology and chemotherapy, and this is still evolving.^4^,^5^ Although most centers prefer surgery for local treatment in Ewing’s sarcoma, mutilating surgery like amputation is not advocated. Radiation therapy remains the mainstay for patients where limb salvage surgery cannot be performed. The results of such patients are reported to be inferior, but it may be attributed to the bias in patient selection.^6^

Treatment of Ewing’s sarcoma is established as a multimodality approach.^1^–^3^ Several studies have reported improved 5-year survival rates for nonmetastatic Ewing’s sarcoma.^3^,^7^–^9^ However, there is paucity of data in the Indian population.^1^ In the context of the limited resources, delayed presentation, inadequate treatment due to socioeconomic factors and lack of adequate follow-up, the results of multimodal management of Ewing’s sarcoma in Indian patients need to be analyzed critically. Therefore, we conducted the present study to analyze the outcome of multimodality treatment of Ewing’s sarcoma of the extremities at a tertiary nonprofit institute over a decade.

## MATERIALS AND METHODS

All patients with proven diagnosis of Ewing’s sarcoma between the period of 1997 and 2007 were noted from the hospital’s cancer registry and those with extremity involvement were identified. Clinical charts of all patients with a diagnosis of Ewing’s sarcoma involving the extremities were reviewed after obtaining institutional review board approval. The patients with confirmed histopathological diagnosis who received treatment at our institute were included in the study. The charts of these patients were analyzed in detail for demographic profile, clinical features, imaging studies, treatment and outcome. The information on the final outcome was obtained from the clinical charts and was complimented with telephone calls in seven patients. The patients were treated by two different oncology teams and the same institutional protocol was adhered to for all the patients.

The diagnosis was established on the basis of histopathological examination and immunohistochemistry studies whenever required. Tumor was designated as small or large based on the tumor size </> 8 cm on available imaging (X-ray, computed tomography [CT] scan, magnetic resonance imaging [MRI]). Metastatic workup included CT scan of the chest, bone scan, bone marrow examination and, more recently, positron emission tomography scan. All patients received four to six cycles of neo adjuvant chemotherapy and six to eight cycles of adjuvant chemotherapy. The regimen of chemotherapy followed was Vincristine, Actinomycin D, Cyclofosfamide alternating with Ifosfamide and Etoposide, at three-weekly intervals.^2^–^4^ Surgery was generally preferred wherever resection with negative margins seemed feasible without significantly compromising form and function of the limb, and the patient had localized (nonmetastatic) disease. Radiotherapy was chosen as the definitive form of local therapy where surgery was not found suitable and added as adjuvant to surgery where histopathological examination revealed close margins or poor necrosis. However, the form of local therapy was also influenced to a great extent by the choice and expectations of the patient and was decided after a detailed discussion between the patient and the primary physician. Local treatment was radiation alone (n = 15), surgery alone (n = 12) and both surgery and radiation (n = 4). Three patients did not receive any local treatment due to multicentricity of the disease. After completion of treatment, the follow-up was 3-monthly for the initial 2 years and 6-monthly thereafter. Every follow-up visit consisted of local and regional examination, CT scan of chest and local imaging (X-rays routinely, complimented by MRI/CT scans where suspicion of a local relapse was present). The functional outcome of the patients was evaluated using the musculoskeletal tumor society (MSTS) scoring system.^10^ Relapse (local or systemic) and disease progression (labeled as appearance of new metastatic lesions/increase in size of the primary lesion) were considered as events. The outcome was correlated with age (less than or more than 10 years), sex, size of tumor (less than or more than 8 cm in the largest dimension), stage at presentation, modality of local treatment (surgery, radiotherapy or a combination of both) and site of relapse (lung or bones). The overall and event-free survivals were evaluated for all patients using the Kepler Miers curve (SPSS).

## RESULTS

This retrospective study includes a total of 137 patients diagnosed as Ewing’s sarcoma family of tumors (ESFT) registered at our institute during 1997 to 2007. Of these, 72 patients received treatment and follow-up for Ewing’s sarcoma at the institute, while the other patients did not report for further management after their initial visit. Thirty-four of these 72 patients had an involvement of the extremity and were included in the present study. The diagnosis was established by review of outside slide/block in 19 patients, a trucut/core biopsy in 11 patients and an open biopsy in four patients.

The age of the patients ranged from 1 to 48 years (median, 22 years; 10 patients <10 years, 24 patients >10 years). Twenty-seven patients (79%) were males. Lower limb was involved in majority of cases (82%), and the most common bone affected was femur (35%). Twenty-nine patients (85%) were Enneking stage II B at presentation while five patients (15%) were Enneking stage III (three pulmonary metastases, two combined pulmonary and skeletal metastases). Sixty-two percent of the patients had a tumor larger than 8 cm in the largest dimension. Radiation alone was the most common mode of local treatment (44%), followed by surgery (35%) and the combination of surgery and radiation (12%).

**Figure 1:**
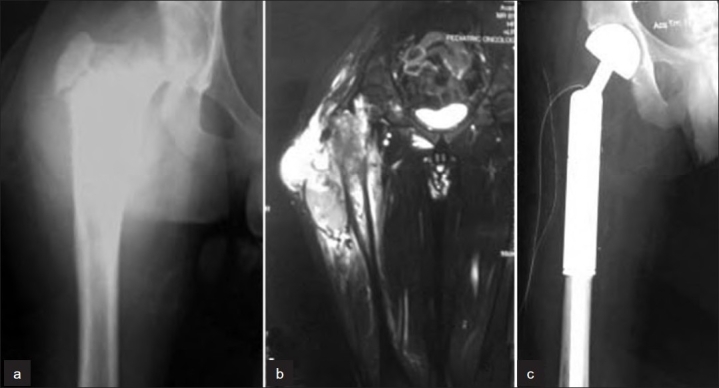
Ewing's sarcoma of the right proximal femur in a 15 yr old male, Enneking stage IIB. (a) X-ray of hip (anteroposterior view) showing the bony destruction with soft tissue mass. T2W1 saggital of hip (b) showing the intramedullary extent of the proximal femur involved along with extracompartmental extension with soft tissue mass. (c) Postoperative radiograph of hip (anteroposterior view) depicting wide resection and reconstruction with modular prosthesis

Pain was the presenting symptom in 30 patients, while swelling as a presenting complaint was seen in 27 patients. Eighteen patients presented more than 3 months after onset of symptoms, of which 15 had been treated as some other disorder for months. All patients received four to six 6 cycles of neo adjuvant chemotherapy and six to eight cycles of adjuvant chemotherapy. Local treatment was radiation alone (n = 15), surgery alone (n = 12) [Figure 1] and both surgery and radiation (n = 4). Three patients did not receive any local treatment due to multicentricity of the disease. The margins of resection were wide (tumor covered by a cuff of normal tissue all around) in 12, marginal (tumor covered only by a capsule/psuedocapsule) in four and intralesional (tumor peeled off its capsule or broken at any place) in none of the patients. The dose of radiation delivered varied between 55 and 60 Gy. At the final follow-up (mean, 26 months; median, 17 months; range, 3–97 months), 24 patients were alive and 10 (eight of these were stage IIB while seven had tumor size >8 cm) had died with disease (at a minimum follow-up of 3 months). A total of 19 events (progression of disease in eight and relapse in 11 patients) occurred in as many patients. Of the 11 patients having a relapse, four had local recurrence, four had distant skeletal metastases and three had pulmonary metastases. Of these 19 patients, eight patients abandoned treatment (lost to follow-up) and the remaining 11 were given palliative treatment in the form of palliative chemotherapy, radiotherapy or supportive care or a combination of two or three of these.

**Figure 2:**
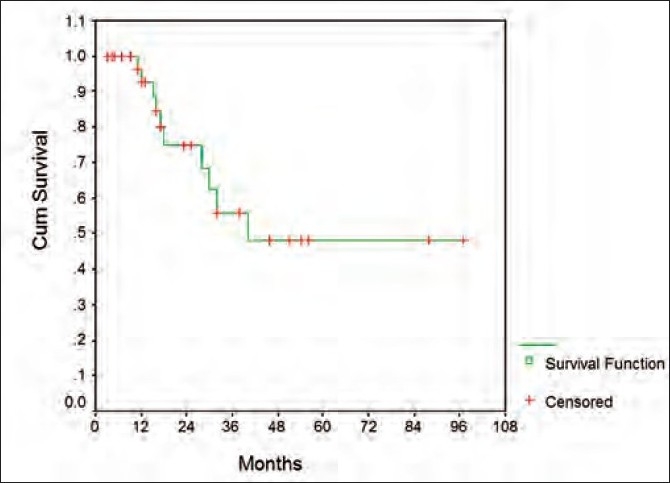
Graph depicting overall survival of patient (see text)

**Figure 3:**
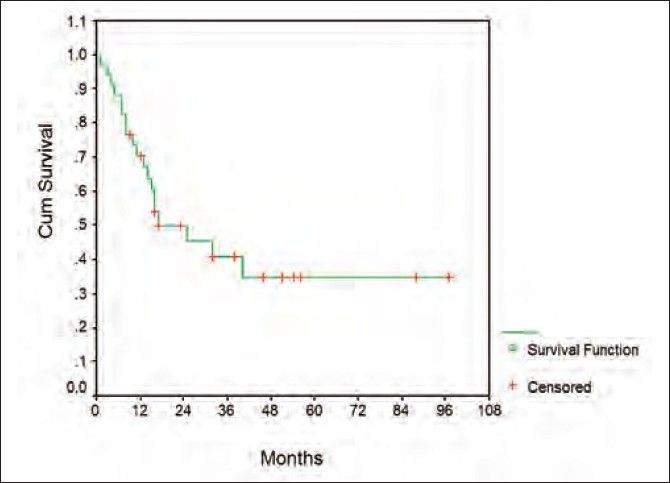
Graph depicting event-free survival of patient (see text)

Overall, the median survival was 40 months while the 5-year survival rate was 47 ± 12% [Figure 2]. The corresponding figures for the event-free survival were 17 months and 34 9% [Figure 3].

**Figure 4:**
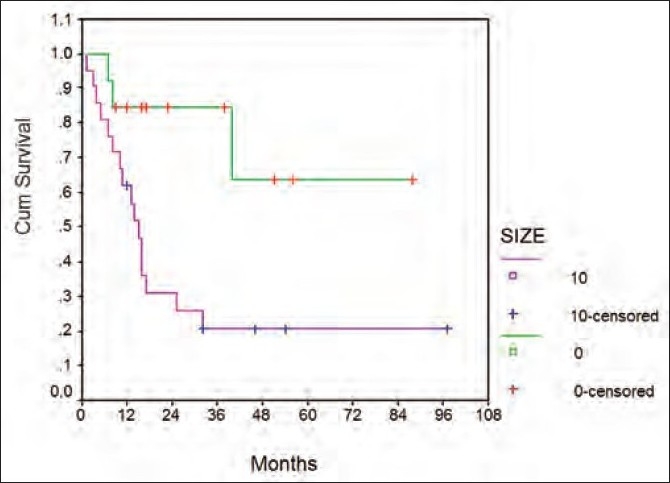
Graph depicting the correlation of tumor size (</>8 cm) with event-free survival (*P* = 0.01)

There was no significant difference in the overall survival of patients when correlated with age (less than or more than 10 years) (*P*=0.08), sex (*P*=0.7), size of tumor (less than or more than 8 cm in the largest dimension) (*P*=0.27), stage at presentation (*P*=0.63), modality of local treatment (surgery, radiotherapy or a combination of both) (*P*=0.59) and site of relapse (lung or bones) (*P*=0.6). Event-free survival on the other hand was significantly affected only by the size of the lesion (univariate analysis) (*P*=0.01) [Figure 4].

The average MSTS score^10^ was found to be satisfactory, better than 16, in all the patients, the average MSTS score being 21. All the patients were satisfied with the functional outcome. None of the patients underwent amputation. The MSTS score did not correlate with the modality of local therapy.

## DISCUSSION

Management of Ewing’s sarcoma has evolved over the last few decades to the present treatment involving multiagent chemotherapy combined with surgery and/or radiotherapy. While this has led to a major improvement in the outcome of localized Ewing’s sarcoma in terms of long-term survival rates,^9^,^11^ the results of metastatic Ewing’s sarcoma are still dismal. Although there is a lot of data in the world literature, the literature on the outcome of Ewing’s sarcoma of extremities in Indian patients is scarce.

The incidence of Ewing’s sarcoma has been reported to be lower in Asian populations as compared with Caucasians.^12^,^13^ In the Indian population, however, it has been reported to be relatively common among malignant primary bone tumors.^14^ Our study on Indian patients reflects the statistics reported in other populations as regards mean age and sex distribution,^15^ and males outnumbered females. Size of tumor has often been reported to be an important predictor of a poor prognosis.^16^ Our study too showed a significant effect of tumor size on event-free survival. Stage at presentation is another important factor predicting survival.^12^ However, the number of patients presenting with metastases in our study is too small to be of any analytical value. In our study, only five of the total 34 patients had metastatic disease at the time of diagnosis, which is comparable to the western world, where the referral system is good and primary care is available very early. However, it is important to realize that these five patients are out of the 34 patients who opted for treatment. It is possible that many patients with metastatic disease did not agree for the treatment when they were told about the prognosis of metastatic disease, making the percentage of metastatic patients in our study relatively low. Modality of local treatment (radiation, surgery or a combination of both) showed no significant effect on the outcome in our patients. Although this agrees with some other studies,^17^,^18^ this is in contradiction with the general consensus now on the choice of modality of local therapy.^2^,^3^,^19^ It is important to realize that the outcome of our patients has been analyzed at the median follow-up of 17 months (range, 8–97 months) and that the results may change at a longer follow-up because Ewing’s sarcoma is known to have late recurrences and, also, second malignancy due to chemoradiation takes longer to manifest.^20^ Although there is a general agreement now for surgical treatment as the choice of local therapy, there may be a bias of selection of patients where the type of local modality is chosen on the basis of age, lifestyle, socioeconomic factors and site and size of tumor. Indeed, because of the heterogeneity of the disease, biologic and treatment variables, it is difficult to analyze the role of local control modality in the treatment of Ewing’s sarcoma.^21^ Hence, the choice of local treatment has to be individualized to the patient.

In our study, the overall survival (OS) and event-free survival (EFS) were noted to be inferior compared with that reported in the world literature, where OS and EFS are reported to be higher.^19^,^22^ While the demographic profile, stage at presentation and the chemotherapy regimen followed in our study were similar to the world literature, a larger proportion of our patients presented with a larger tumor size (although we included only extremity Ewing’s sarcoma, which are supposed to have smaller sizes at presentation). Sixty-two percent of our patients presented with a larger tumor size, which is higher compared with the world literature.^2^,^17^ This may explain a poorer outcome in our patients.

Functional implications are often a major deterrent for surgery as the local treatment modality. While surgery would mostly mean amputation in the earlier era, most patients are now amenable to limb salvage surgery. Hence, the role of surgery has increased in the treatment of Ewing’s sarcoma over time.^19^,^23^ The functional outcome was similar in patients in the surgery group as compared with that in the radiation group. The follow-up in our study is too short for the late effects of radiation (including radiation-induced sarcoma and fracture/nonunions) to manifest.

Management of patients in India deserves special attention as we have a very diverse population. There are financial constraints, poor education, poor socioeconomic status and availability of alternative therapy that have a significant impact on the overall outcome.^24^ As is evident from our study, a large majority of patients received improper treatment or were treated as some other disease before they presented to our center. We also noted that 72 out of a total of 137 patients did not choose to get any treatment at our institute. Moreover, of the 19 patients having an event, eight patients abandoned treatment. While it is possible that some of these patients took treatment at other centers, other reasons like lack of resources, social factors and availability of alternative medicines cannot be neglected in the Indian scenario. We admit that our study is limited by a relatively short follow-up and smaller number of patients, and a larger series with a longer follow-up is desirable to assess these factors in greater detail.

## CONCLUSION

In spite of a similar demographic profile, stage at presentation and local and systemic treatment regimen, the outcome of Ewing's sarcoma in our patients does not parallel the improvement reported in the literature. Although multimodal management has improved the outlook for these patients, larger multicentric studies are required in the Indian context too address the issues of late presentation, poor follow-up and cost constraints
